# Deciphering the Calcium Code: A Review of Calcium Activity Analysis Methods Employed to Identify Meaningful Activity in Early Neural Development

**DOI:** 10.3390/biom14010138

**Published:** 2024-01-22

**Authors:** Sudip Paudel, Michelle Yue, Rithvik Nalamalapu, Margaret S. Saha

**Affiliations:** 1 Wyss Institute, Harvard University, Boston, MA 02215, USA; sudip.paudel@wyss.harvard.edu (S.P.); michelle.yue@wyss.harvard.edu (M.Y.); 2 School of Medicine, Virginia Commonwealth University, Richmond, VA 23298, USA; nalamalapurr@vcu.edu; 3 Biology Department|William & Mary, Williamsburg, VA 23185, USA

**Keywords:** calcium, embryo, neural, spikes, development, review

## Abstract

The intracellular and intercellular flux of calcium ions represents an ancient and universal mode of signaling that regulates an extensive array of cellular processes. Evidence for the central role of calcium signaling includes various techniques that allow the visualization of calcium activity in living cells. While extensively investigated in mature cells, calcium activity is equally important in developing cells, particularly the embryonic nervous system where it has been implicated in a wide variety array of determinative events. However, unlike in mature cells, where the calcium dynamics display regular, predictable patterns, calcium activity in developing systems is far more sporadic, irregular, and diverse. This renders the ability to assess calcium activity in a consistent manner extremely challenging, challenges reflected in the diversity of methods employed to analyze calcium activity in neural development. Here we review the wide array of calcium detection and analysis methods used across studies, limiting the extent to which they can be comparatively analyzed. The goal is to provide investigators not only with an overview of calcium activity analysis techniques currently available, but also to offer suggestions for future work and standardization to enable informative comparative evaluations of this fundamental and important process in neural development.

## 1. Introduction

The flux of calcium ions within and between cells constitutes an ancient, ubiquitous, complex, yet highly efficient signaling pathway tightly regulated by an array of secondary messengers and accessory proteins [1,2]. Although most extensively studied for its essential role in regulating neurotransmission, calcium activity plays a key role in virtually every physiological process and tissue type, ranging from the immune response and muscular control to the stress response, wound healing, cardiac myocyte function, brain homeostasis, cell proliferation, cell senescence, and more [3,4,5,6,7,8,9,10,11,12]. In addition to the numerous roles calcium plays in the mature adult organism, calcium also acts as a critical and essential signaling molecule in developing systems [13]. While its role in the very initial stage of development, namely fertilization, has been extensively investigated in several model systems, calcium dynamics are also known to play a role in developmental processes from early cleavage through the onset of mature action potential-driven neurotransmission. Post-fertilization, calcium activity has been implicated in cell cycle dynamics and the control of cleavage, neural induction, and neuronal phenotype determination [13,14]. In fact, the role of calcium has been deemed sufficiently critical to so many developmental processes that investigators have employed the phrase “calcium code” for the array of activities regulating these processes within the embryo, a phrase originally utilized for adult neuronal physiology. Additionally, we provide the citations for the explanation of the elusive “calcium code [15,16,17]”.

However much of the evidence for the role of calcium signaling in development derives from molecular genetic data, correlating the expression of proteins associated with calcium signaling with key developmental events and demonstrating that perturbing these genes negatively impacts developmental processes. While these data suggest that calcium dynamics are important in many processes, they are often not accompanied by any type of in vivo calcium imaging; thus, they are unable to demonstrate calcium signaling directly in the processes they implicate. Although fewer in number, there are several excellent studies that do investigate the role of calcium activity in development using in vivo calcium imaging, but they tend to use completely different imaging and analysis methods, making comparisons between studies and integration of knowledge across studies quite challenging. Given that several comprehensive reviews on the role of calcium activity in development exist (see [18,19,20,21]), the goal of this review is to focus on the methodological aspects of in vivo calcium imaging, specifically the techniques used for analysis by summarizing, comparing, and contrasting the techniques that have been historically used to study calcium. In particular, cleavage stages, neural induction, neuronal subtype formation, and neurotransmitter phenotype specification are explored. Overall, this review reveals how a wide array of calcium detection and analysis methods are used across studies, limiting the extent to which the studies can be comparatively analyzed. The goal is to provide investigators not only with an overview of the range of currently employed calcium activity analysis techniques, but also suggestions for future work and standardization to enable informative comparative evaluations.

## 2. Background

During calcium signaling events, influxes of calcium enter into the cytoplasm from either intracellular stores (e.g., from the endoplasmic reticulum) or the extracellular matrix, transiently increasing intracellular calcium concentrations; this elevated free, cytosolic calcium can then activate ion channels or act as a second messenger to indirectly activate other signaling pathways (e.g., G protein-coupled receptors and downstream effectors molecules) to regulate gene expression [22,23]. Although cells exhibit a range of calcium activity dynamics both temporally and spatially, during early neural development embryos display two major types of spontaneous calcium fluxes, namely spikes and waves [24]. A spike is a high-amplitude transient increase and subsequent decrease in intracellular calcium (Ca^2+^) concentration, which occurs through activation of voltage-gated calcium channels, including L-type channels or IP_3_-mediated release from the endoplasmic reticulum. Spikes exist for a relatively short duration (approximately 10 s or less) and are restricted to single cells [25,26]. Contrastingly, waves are characterized by low-amplitude changes in intracellular calcium concentration. Compared to calcium spikes, they have lower frequencies and exist for longer durations and involve multiple cells. Calcium waves first originate upon activation of IP_3_ receptors or ryanodine receptors under resting condition, with calcium activity propagating to neighboring cells through gap junctions [27]. When this propagation leads to multiple cells, this is defined as a wave. These inter-cellular waves are triggered by variety of stimuli including release of calcium ions from internal store, extracellular messengers via paracrine signaling [28], and predominantly via internal messengers that move through gap junctions [27]. While gap junctions are widely expressed during embryonic development, these are also found in adult tissues as well such as epithelia, nerves, cardiac (heart) muscle, and smooth muscle (such as that of the intestines). They are a means of cell-cell contact and communication, calcium signaling being one of them [29]. Both spikes and waves are known to occur throughout all embryonic cells and developing tissues [14].

These types of calcium signaling events can be visualized by using calcium imaging techniques such as using calcium-sensitive marker dyes (e.g., Fluo-4 or Fura-2) or genetically encoded calcium indicators (GECIs) (e.g., GCaMP or RCaMP). Upon binding with free cytosolic calcium ions, these calcium-sensitive dye or GECI markers change configuration and fluoresce, enabling calcium visualization through fluorescent microscopy. While relative fluorescent values (RFU) vary across different markers, the measured fluorescence levels for each calcium-sensitive marker and intracellular calcium levels can be correlated and quantified, with increased fluorescence indicating increased intracellular calcium. GCaMP6s (a GECI), for instance, has a 3-fold higher affinity for calcium than GCaMP3 (a GECI), consequently producing higher fluorescence values than GCaMP3 for any specific intracellular calcium concentration [30]. Additionally, these indicators have been shown to exert unwanted, deleterious effects on cells [31]. Despite their limitations, these markers provide an inferential yet valuable metric for in vivo calcium signaling and function [32,33,34,35,36].

**Figure 1:**
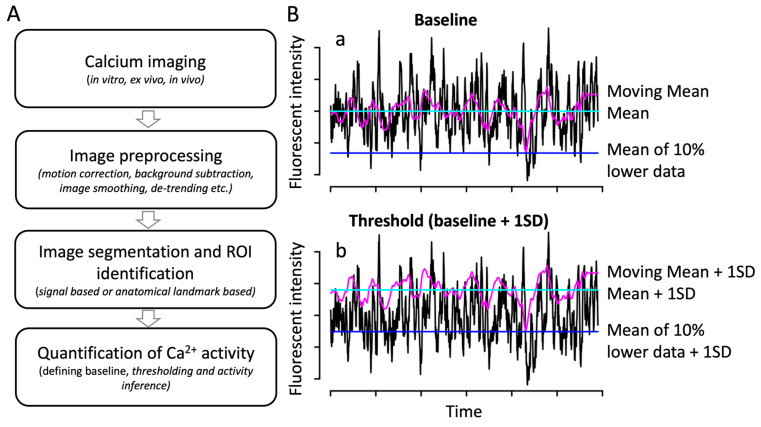
Typical outline of calcium image processing and subsequent data analysis pipeline: calcium imaging; image pre-processing, which includes but not limited to motion correction, background subtraction, image smoothing, data de-trending; image segmentation and ROI identification; and quantification of calcium activity (**A**). Quantification of Ca^2+^ activity (**B**) with an example of impact of application of three different baselines: moving mean of the time series (magenta); mean of time series (cyan); and mean of 10% of lower values of the time series (blue) (**Ba**) while using even the same threshold of 1 standard deviation (SD) of the time series to define what constitute a calcium activity event (**Bb**).

In general, the pipeline used to analyze calcium imaging data at cellular resolution is comprised of several steps: (1) image processing, which includes correction of motion or drift during an imaging session, background correction, and image segmentation to identify regions of interest (ROIs), (2) tracking of cells over time to obtain time series of cellular calcium, (3) quantification of calcium activity, and (4) spatiotemporal pattern analysis (Figure 1A). While this pattern of steps involved in the calcium image analysis pipeline is generally consistent across studies, each of these steps within the pipeline is highly modular and is very different depending on the given study.

**Figure 2:**
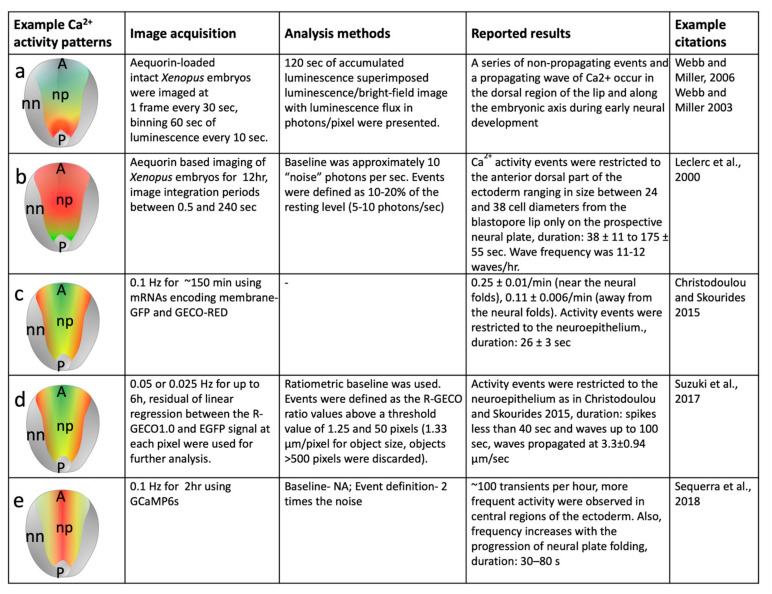
Heatmap drawings (**a**–**e**) showing examples of discrepancies in reported spatial patterns of calcium activity during early neural development of an example model system (*Xenopus* neural plate). The neural plate (np) is depicted anterior-posteriorly (A/P). Calcium activity, from highest levels to lowest levels, are indicated by the following colors: red, yellow, green, and blue. Gray color indicates lowest levels of calcium activity, which is usually in non-neural (nn) tissue. While some studies have reported higher levels of calcium activity near the blastopore areas (**a**) [18,39], others have shown higher levels in the neural plate (**b**,**e**) [40,41] or neural ridge areas (**c**,**e**) [41,42,43]. One of the reasons for this difference could be due to application of differential imaging parameters as well as calcium activity analysis methods. Note that studies cited in this figure are only the representative studies. For further detail see Supplementary Table S1.

For instance, studies generally quantify changes in fluorescent measurements of a calcium reporter (∆F) in order to study calcium transients using time series data collected; however, techniques used to define background signal (i.e., techniques for signal preprocessing) and define what baseline measurements are may dramatically impact results and conclusions of a study [1Ba]. Furthermore, in order to determine whether or not a given cell has calcium activity at any given point in time, studies implement threshold fluorescent values when analyzing calcium activity time series data [1Bb]. Choosing an appropriate activity threshold for analysis, especially when calcium activity traces in the time series data are irregular and complex (i.e., composed of multiple frequencies and amplitudes), may have a significant impact on the results and conclusions of a study (Figure 2). In particular, irregular and complex calcium activity is more prevalent in certain cell types, such as embryonic presynaptic neuronal cells given the lack of clear periodicity in Ca^2+^ influxes [37].

Thus, factors in addition to image acquisition rate and calcium indicator type, such as the lack of standardization practices for determining activity threshold for calcium activity, may cause studies—seemingly similar in their experimental techniques—to report dramatically different conclusions (reviewed in [36,38]). This review identifies and analyzes the various techniques used to analyze calcium activity during development, revealing a wide array of different methods used for calcium visualization and image acquisition, background definition, baseline definition, and calcium activity thresholding, which may have important implications on the accuracy and reliability of conclusions drawn by studies about embryonic calcium activity (Supplementary Data, Table S1).

## 3. Calcium Activity Detection Methods in Cleavage Stages

### 3.1. Early Cleavage Stages

After fertilization of an egg by a sperm, the resulting zygote undergoes a series of cell divisions, called cleavages, to form a morula and subsequently a blastula (i.e., ball of cells with a hollow blastocoel space within it). Multiple studies using several model systems and calcium detection techniques have established that calcium transients are observed at each cleavage during the early cleavage stages, implicating calcium’s involvement in blastula formation. Early studies by Fluck et al., collected calcium imaging data in medaka fish zygotes using the aequorin calcium indicator (a luminescent reporter) and imaging with a photon detector, demonstrating that embryos exhibit two successive calcium waves along the cleavage furrow at the first three cleavages [44,45]. The researchers quantitatively analyzed in vivo imaging data through the quantification of photons detected at each pixel and found that two calcium transients are associated with movement of the cleavage furrow, hypothesizing that the first, narrower wave is necessary for furrow growth (regulating actomyosin shortening) and the second, broader wave is necessary to produce a new cleavage furrow. These studies did not conduct calcium activity analysis of images by defining calcium transients (i.e., spikes and waves); they estimated peak calcium levels of cleavage furrow waves as being 5–8 µM by finding the ratio of light per active cell volume over light per resting cell volume (baseline), normalizing to a baseline cytosolic calcium level of 0.14 µM, and using a graph correlating aequorin luminescence with calcium levels from Blinks, which, at midrange, shows that f-aequorin varies with calcium to the 2.5th power [44,45,46].

Using a different model organism but similar calcium detection technique, Webb et al., assessed calcium activity imaging data in zebrafish embryos during cleavage stages using the f-aequorin calcium indicator (a luminescent reporter) and imaged with a photon detector [47]. While this study analyzed the resulting data by quantifying the number of photons detected at each pixel, the authors manually identified the calcium transients present in its images. Webb et al., observed the same two types of calcium transients as those found in Fluck et al., in addition to uncovering that these waves occur in a weaker form in parthogenetically activated embryos, supporting the conclusion in Fluck et al., that calcium is involved in early cleavage stages. To estimate peak calcium levels during calcium transients observed, Webb et al., similar to Fluck et al., calculated relative photon intensities per volume using the ratio of photons per active cell volume over the photons at resting cell volume (baseline), which was determined by calculating photons per resting volume in an area where there is no calcium activity [47]. Using their assumption that the resting level of calcium in fertilized zebrafish eggs was 160 nM and their assumption that f-aequorin varies with calcium to the second power, the study determined that calcium levels rise 670 nM in the furrow positioning signal, 650 nM in furrow propagation signal, and 437 nM in the cleavage furrow deepening signal. Differences in calcium analysis techniques (i.e., baseline definition and correlation between f-aequorin and calcium levels) may contribute to discrepancies in peak calcium levels during transients determined by Fluck et al. and Webb et al. [44,45,47].

Using a different model organism and calcium marker from both Fluck et al. and Webb et al. to investigate calcium signaling during early cleavage stages, Muto et al., imaged calcium activity in *Xenopus laevis* embryos using the fluorescent calcium reporter, calcium green-1 dextran (CaGr) [48]. Similar to Fluck et al. and Webb et al., this study manually identified the presence of calcium transients in its images and found similar calcium transients along the cleavage furrow. In particular, Muto et al., determined the maximum increase in fluorescence to be 1.64 ± 0.20-fold by comparing the average fluorescence intensity in a region (that was 0.5% of the total cell area) by the first cleavage furrow to the fluorescence intensity at baseline before the calcium transient was present. Muto et al., also determined that these transients were inhibited by heparin, an inositol 1,4,5-triphosphate receptor antagonist. This led to the study’s conclusion that the inositol 1,4,5-triphosphate receptor mediates calcium signaling during cleavage stages of development [48].

In a separate study, Noguchi and Mabuchi imaged calcium activity at the first cleavage of *Xenopus laevis* embryos using calcium green-1 dextran (similar to Muto et al.) and the calcium-insensitive rhodamine dextran (a volume indicator) or, for simultaneous cortex imaging, calcium green-1 dextran and CNF (5-(and 6-)-carboxynaphthofluorescein succinimidyl ester) dextran (a volume indicator) [2]. Time-lapse imaging of calcium waves was performed using a confocal laser scanning microscope, with fluorescence intensities of calcium green-1 dextran and rhodamine dextran (or CNF dextran) made equal at baseline. Time-lapse data were processed using the LSM 510 software, and increases in fluorescence intensity were determined by subtracting the fluorescence intensity of rhodamine dextran (or CNf dextran) from the fluorescence intensity of calcium green-1 dextran.

In addition, Noguchi and Mabuchi imaged smaller calcium transients termed “calcium puffs” and “calcium blips” using the Fluo-4 calcium indicator and confocal laser scanning microscopy [2]. Confocal serial line-scans were performed and increases in fluorescence intensity in the animal hemisphere were calculated using F_increase_ = F − F_rest_, with baseline F_rest_ defined as the fluorescence intensity in the same location prior to the initiation of the calcium puff or blip. When investigating calcium puffs and blips at the cleavage furrow growing end, thirty frames of imaging data were collected over 23.4 s (at a rate of 0.78 s per frame), and fluorescence intensity was calculated frame-wise using F_increase_ = F_n_ − F_n−1_, with F_n_ defined as the fluorescence of Fluo-4 at frame n, and with F_n−1_ defined as the fluorescence of Fluo-4 at frame n − 1. Overall, the study determined that calcium puffs lasted 150–500 milliseconds and were 4.3 ± 0.65 μm in diameter at half-maximum intensity, while calcium blips lasted less than milliseconds and were 1.8 ± 0.45 μm in diameter at half-maximum intensity [2].

Furthermore, Noguchi and Mabuchi demonstrated that the calcium transients occurred after cleavage furrow formation and that cleavage itself was not affected by injection of dibromo-BAPTA or EGTA, which suppressed the calcium transients. Through this observation, the study supported its conclusion that the calcium transients observed were likely not involved in cytokinesis. Because the second transient observed propagated along the border of the new and old membrane, the study hypothesized that it may be involved in adhesion between blastomeres [2].

While calcium signaling during early cleavage stages of development has been observed in several studies, the specific role of calcium in cleavage furrow formation has been contested [2,44,45,47,49]. In particular, Noguchi and Mabuchi [2] disagreed with many existing studies by suggesting that calcium signaling does not play a role in cleavage formation and instead may be involved in blastomere adhesion [47,48,49]. This conclusion was based on their two observations: (1) the calcium waves appeared in a region of egg that is spatially separated from the contractile ring after formation and contraction of the contractile ring, and addition of new membranes in the cleavage furrow, and (2) suppression of calcium activity using potent inhibitors dibromoBAPTA or EGTA did not affect the cleavage formation. It has been suggested that increased intracellular calcium ions is required for calmodulin mediated phosphorylation of myosin regulatory light chain, which contributes to contractile ring formation (reviewed in [50]). Also, when cleavage is blocked using colchicine, IP3 levels, which mobilize Ca^2+^ from storage organelles, increase at the expected time after insemination (reviewed in [51]). IP3 activation usually results in increases in the intracellular Ca^2+^ concentrations. However, Noguchi and Mabuchi showed that suppression of Ca^2+^ activity did not impact contractile ring formation and cleavage furrow growth [2]. It is possible that there is a molecular clock that activates IP3 pathway but subsequent increase in Ca^2+^ concentration may not be required for cytokinesis [51] or the amount of phosphorylated myosin may increase somewhere other than the cleavage furrow region [2].

Overall, studies investigating the role of calcium activity in early cleavage have been conducted in several model organisms (e.g., medaka fish, zebrafish, and *Xenopus laevis*) using the aequorin luminescent reporter calcium indicator and fluorescent calcium reporter, Calcium Green-1 dextran. An overarching limitation of these studies is their lack of rigorous, automated calcium transient analysis as well as their variation in defining baseline fluorescence intensities and fluorescence intensity increases, demonstrating a lack of standardization in calcium imaging analysis techniques across existing literature.

### 3.2. Blastula and Gastrula Stages

To investigate the role of Wnt-5/*pipetail* on the Wnt/β-catenin pathway, Westfall et al., imaged calcium activity in zebrafish embryos at 0.1 to 0.06 Hz for 45- to 75-min imaging sessions, 10 to 20 min after pharmacological treatment with XeC, a membrane-permeable blocker of IP_3_-mediated calcium [52]. In order to determine calcium fluxes, Westfall et al., used subtractive analog analysis, identifying a significant increase in Fura-2 fluorescence intensity with respect to Texas-Red fluorescence intensity. The study found that loss of function of the Wnt-5 protein resulted in reduction of calcium signaling and inhibition of the Wnt/β-catenin pathway, leading to the conclusion that Wnt-5/*pipetail* is involved in cell movement and modulates the Wnt/β-catenin pathway through calcium release [52].

Furthermore, Lyman Gingerich et al., imaged calcium activity in zebrafish embryos from the 32-cell stage to the 128-cell stage (blastula stages) [53]. This study used the ratio-metric calcium indicator fura-2-dextran to image calcium activity with an imaging rate of four frames per minute over 65 min. Additionally, this study analyzed its calcium imaging data using methods described in Slusarski et al. [54] and Westfall et al. [52,55] which determined changes in calcium activity using subtractive analog analysis (similar to Westfall et al.) and defined a calcium transient as a cell-sized feature that exhibited significant increase in fluorescence intensity [54,55,56]. As part of this study, Lyman Gingerich et al., found that *hec*-maternal effect mutant zebrafish embryos had a 5- to 10-fold higher number of calcium transients in the blastula envelope layer compared to wild-type embryos. Additionally, this study demonstrated how inhibition of calcium release in these *hec*-mutants caused abnormal dorsal gene expression. Overall, the results of this study support that during cleavage stages of development, the *hecate* gene is involved in calcium release, impacting dorsal gene expression.

In addition, Eno et al., imaged calcium activity in zebrafish embryos during the cleavage stage using fura-2-dextran (similar to Lyman Gingerich et al., or Oregon green 488 BAPTA dextran to determine how calcium transients impact ribonucleoparticle recruitment to furrows during cell cleavage [53,57]. This study imaged calcium activity at 0.06 Hz for a total of 1500 frames, and at each time point, the Rhodamine dextran intensity was subtracted from OGB intensity as a form of baseline subtraction. While this study stated that calcium data were analyzed using the AxoGraph software, it did not state how calcium transients were defined in the analysis. Through this study, it was revealed that *nebel* mutants—associated with reduced, slow calcium waves during cleavage—displayed medially bundled microtubules, supporting the conclusion that the calcium transients identified during cleavage facilitate germ plasm ribonucleoparticle recruitment to the furrow by providing medial-to-distal directionality [57].

Using the same model organism but a different calcium detection method compared to Eno et al., Mizuno et al., imaged calcium activity in zebrafish embryos during the cleavage and blastula stages by creating transgenic zebrafish lines with constitutively active hspa8-driven expression of the YC2.60 ratio-metric calcium indicator [49]. In particular, Mizuno et al., used time-lapse images from two-photon or line-scanning confocal microscopy and analyzed fluorescence resonance energy transfer (FRET) signal without applying a threshold. The study revealed that one wave synchronized to cleavage is exhibited by embryos and that calcium activity begins at the animal pole, propagating across the future cleavage furrow [49].

Additionally, Ma et al., investigated the role of calcium signaling during early development by imaging zebrafish embryos at animal pole during the blastula and gastrula stages using the calcium-green dextran fluorescent calcium reporter and f-aequorin luminescent reporter [58]. In order to quantify calcium activity, Ma et al., used an automated algorithm that applied the Tensor Voting Framework to identify cell centers and boundaries of calcium signaling clusters. This study reported that pre-gastrulation embryos exhibit aperiodic calcium activity primarily in superficial epithelial cells, but post-mid-blastula transition, there is a dorsal bias in calcium activity for about one hour [58].

In a separate study, Zhang et al., attempted to determine the role of calcium transients in apical-basolateral thinning during the formation of the enveloping layer (EVL) [59]. This was performed by imaging calcium activity in zebrafish embryos at the blastula stage using a combination of calcium green-1 dextran and rhodamine B isothiocyanate-dextran. Images were taken for 45 min at 0.25 Hz with manual identification of calcium transients instead of quantitative ratio-metric analysis. Additionally, Zhang et al., used luminescent *f*-aequorin calcium indicator to investigate spatial patterns in calcium activity generated by the EVL during the blastula period. Similar to Ma et al., Zhang et al., used an automated algorithm that applied the Tensor Voting Framework to identify cell centers and boundaries of calcium signaling clusters to analyze the *f*-aequorin-generated data. Zhang et al., found that treatment with the Wnt-5A agonist upregulated EVL-restricted calcium transients and increased shape change of EVL cells, while treatment with antagonists thapsigargin or U73122 disrupted calcium transients and EVL cell thinning. Through these observations, the study concluded that calcium transients in the EVL play a role in initiating the apical-basolateral thinning of EVL cells [59].

In a study investigating calcium waves during gastrulation and segmentation, Gilland et al., imaged yolk cells in zebrafish embryos from blastula through segmentation stages using the f-aequorin luminescent calcium reporter for calcium visualization, constructing image sequences with data integration between 0.5 and 240 s [60]. This study detected calcium transients by identifying time points where the number of photons per pixel per second was higher than at baseline (which was established at 10 “noise” photons per second) without implementing a threshold. Using these methods, Gilland et al., identified a quiet period where no calcium activity is present at the beginning of gastrulation with subsequent rhythmic calcium waves initiated in the dorsal midline pacemaker or wave initiation sites propagating around the blastoderm margin every 5–10 min. Furthermore, the study revealed that after epiboly, calcium waves were mainly initiated at a dorsal locus incorporated into the tailbud. This led to the study’s conclusion that calcium waves may be involved in axial patterning [60].

Using similar image analysis techniques as Gilland et al., Leclerc et al., investigated calcium signaling patterns during early embryogenesis by imaging the dorsal ectoderm of *Xenopus* embryos during the blastula and gastrula stages using the f-aequorin calcium indicator [40]. While this study set its baseline for calcium signal analysis as approximately 10 “noise” photons per second, it used manual detection to identify calcium waves in its time-lapse images. In particular, this study exclusively observed calcium waves in the anterior dorsal aspect of the ectoderm and found that the wave frequency increased between stages 9 and 11 (gastrulation). It also found that inhibition of calcium transients with the L-type voltage sensitive calcium channel antagonist R(+)BayK 8644 led to severe defects in the formation of the anterior nervous system, supporting its conclusion that calcium transients may play a critical role in proper nervous system development [40].

In another study aiming to understand calcium signaling during early vertebrate embryogenesis, Reinhard et al., imaged zebrafish embryos at the animal pole during the blastula and gastrula stages using the calcium-green dextran fluorescent calcium reporter and NuCa-green nuclear calcium reporter [61]. The study imaged calcium-green dextran at 0.1 Hz and NuCa-green at 0.2 Hz for 40 to 150 min. To analyze the images of calcium-green dextran fluorescence, the study used three different image subtraction methods and manually identified calcium spikes. Additionally, to analyze the images of NuCa-green, the study defined calcium spikes as increases in dF/F values greater than 16% in 10 s and a fall of less than or equal to 8% in 10 s with a total duration of at most 75 s. This study found that calcium spikes remain restricted to the envelope layer until the end of the blastula stage and occur at an even distribution across the embryo [61].

Yuen et al., also imaged calcium activity in the blastula and gastrula stages of zebrafish embryos in their investigation of calcium waves occurring in the external yolk syncytial layer. In particular, this study used embryos injected with the bioluminescent *f-aequorin* as well as the fluorescent calcium green-1 dextran calcium with rhodamine dextran. While both reporters were used in the study’s experiments, *f-aequorin* data were primarily used to calculate wave velocities in the yolk syncytial layer. In the study, calcium transients in both the yolk syncytial layer nuclei and external yolk syncytial layer cytoplasm were defined as an increase in fluorescent intensity greater than 20% of the baseline rhodamine dextran signal in an approximately 9 µm region of interest; however, the baseline was not explicitly stated in the study’s experimental design. Overall, Yuen et al., found that there was a dorsal bias in the initiation and propagation of calcium waves and that treatment with an IP_3_R antagonist and SERCA-pump inhibitor led to disruption of calcium waves, suggesting that these calcium waves are likely released from IP_3_Rs in the perinuclear ER [62].

In another study imaging calcium activity in zebrafish embryos, Chen et al., created the *Tg[βactin2:GCaMP6s]^st1351^* and *Tg[βactin2:GCaMP6s]^st1352^* zebrafish transgenic lines, imaging calcium activity in them from cleavage to gastrulation using the GCaMP6s calcium reporter [63]. After defining calcium activity as anything above a threshold value of 0.4–0.5 of the dF/F values, calculated by the formula dF/F = (F − F0)/F0, where F is the current fluorescent value and F0 is the mean of all of the fluorescent values along the timeseries for that particular pixel. Using this definition of calcium transients, the study found a dorsal bias in calcium signaling after the mid-blastula transition, consistent with experimental results in Ma et al., (a study where calcium spikes were manually identified). Finally, Chen et al., found that an upregulation of the Nodal protein resulted in increased calcium transient frequency and duration [63].

In a different model organism, Créton et al., imaged calcium activity in *Drosophila* embryos at stage 5 (cellularization stage) using calcium-green dextran and rhodamine dextran. This imaging study was not conducted with a cellular resolution, meaning that it was not able to identify calcium transients at the cellular level unlike most other studies discussed. Instead, this study calculated ratios of calcium concentrations between the dorsal and ventral regions by measuring ratio-metric values of calcium green dextran fluorescence to Texas Red dextran, finding that inhibition of calcium activity affects the amnio serosa (dorsal-most region of the embryo). This led them to the study’s conclusion that calcium signaling plays a role in specification of the dorsal region of the embryo [64].

Créton et al., imaged Zebrafish embryos starting at cleavage stage to segmentation (>11 hpf) for luminescence imaging and stage 4–7 embryos for fluorescent imaging [65]. In this study, they used R-aequorin or h-aequorin for ratiometric imaging and calcium green–1 dextran and Texas red for fluorescent imaging. In order to quantify the luminescence images, the luminescence value was accumulated continuously with 15-min counts centered over each time point for 6 h and the baseline was defined as luminescence twice the resting level luminescence from BAPTA buffered droplets containing comparable levels of free Ca^2+^ and of aequorin droplet dummies. A threshold of 200% of the baseline that lasts 10–200 s (up to 20 min) to define what constitutes a calcium activity event. However, for fluorescent imaging, the calcium green/Texas red ratio was color-coded in a blue to red (ratio of 1.0–1.1), in which red represented the calcium concentrations. They found that on average, the dorsal region has a 9% higher ratio than the ventral region. Also, they suppressed calcium activity by injecting a calcium chelator BAPTA at 1-cell stage. They found that this suppression resulted in impaired eyes, heart, differentiation patterns, and overall development [65].

Also using *Drosophila* embryos, Markova et al., imaged calcium activity in the epithelial layer to study calcium signaling during early tissue morphogenesis [66]. In particular, this study imaged calcium activity beginning at stage 5 (cellularization stage) to stage 10 (end of germ band elongation) using either calcium green dextran or the GECI GCaMP3 at a rate between 1 to 0.1 Hz. Contrasting previously described studies, Markova et al., used a standard deviation (SD)-based approach, where a calcium spike was defined as an increase in fluorescent intensity greater than one standard deviation of the fluorescent signal occurring in the entire cell area. Using this spike definition, the study determined that calcium activity is not restricted to specific tissue precursors (as suggested by Créton et al., but rather increases significantly in the dorsal, ventral, and lateral embryonic regions [66]. Additionally, Markova et al., demonstrated that increases in spike frequency occur during periods of fast tissue remodeling, such as germ band convergent extension and mesoderm invagination. Notably, significantly higher frequency (20 per embryo) of spikes of shorter length (25 s) were identified in embryos expressing calcium green dextran compared to embryos expressing the GECI GCaMP3 (which exhibited three spikes per embryo, 50 s long). Importantly, since this study is observational, it cannot be used to establish a causal effect between calcium spikes and tissue remodeling [66].

Mikhaleva et al., imaged *Oikopleura dioica* embryos starting from cleavage to early tailbud stage using both Fluo4 and GCaMP6s at an acquisition rate of 4 Hz [67]. In this study, they defined a baseline as the average of the fluorescence in 50 frames prior to each transient and the transients were identified by fitting a linear curve. They found that the activity event duration was about 3 s and the inter-peak interval at gastrula stage was approximately 2.54 s. By blocking divalent cations and using T-type Ca^2+^ channel blocker mibefradil. they reported that these treatments result in reduced gastrulation success and disrupted embryonic development [67].

Overall, an array of studies using several model systems (e.g., medaka fish, zebrafish, *Xenopus laevis*, and *Drosophila*) and calcium visualizers (e.g., aequorin, calcium-green dextran, fura-dextran, Oregon Green 488 BAPTA dextran, YC2.60 ratiometric calcium indicator, Texas red lineage marker, and GCaMP GECIs) have established that calcium plays important roles in the early cleavage, blastula, and gastrula stages of development. In particular, future efforts should be made to analyze calcium using quantitative ratio-metric methods rather than manually. Additionally, it is essential for future studies to explicitly and elaborately share imaging rates, how baselines are defined, what normalization techniques are used, and how calcium transients are defined (i.e., what threshold values are used) when analyzing calcium signaling data so that standardized methods for calcium imaging analysis can be established to enable more robust comparisons and contrasts across studies.

## 4. Calcium Activity Detection Methods during Neural Induction

It is during the process of gastrulation that the central nervous system forms from the dorsal ectoderm. Following the blastula stage, a developing embryo undergoes gastrulation, in which a coordinated movement of cells forms the three germ layers: endoderm, mesoderm, and ectoderm. Subsequently, during neurulation, a neural plate is formed. As the neuroepithelial cells in the neural plate change shape, the edges of the plate fold upward (neural fold stage), producing an infolding that seals to form a hollow epithelial tube on the dorsal side of the embryo (i.e., the neural tube). This neural tube eventually gives rise to the brain and spinal cord of the organism.

Neural tube formation is orchestrated by a complex interaction network of many signaling molecules and transcription factors involving around 300 genes (e.g., calcium, bone morphogenetic proteins (BMPs), trophic factors such as brain-derived neurotrophic factor (BDNF), epidermal growth factor (EGF) and insulin-like growth factor (IGF), sonic hedgehog (Shh), and wingless-related integration site (Wnt) [68,69]. While a substantial body of work on neural induction comes from lower vertebrates, several studies contend that the underlying molecular mechanism of neural induction is highly evolutionarily conserved across organisms [70,71,72,73]. Furthermore, while a complex network of signaling molecules and transcription factors orchestrate neural induction, studies using vertebrate and invertebrate model systems have underscored the importance of calcium activity in this process. Calcium’s involvement in neural tube formation was first discovered by Smedley and Stanisstreet when they used electron microscopy to demonstrate that rat embryos cultured in medium with varying calcium antagonists such as papaverine, D-600, and TMB-8 at various concentrations resulted in cephalic neural folds that were elevated but not closed to form hollow neural tubes [74]. With recent advancements in imaging tools, studies have elucidated various spatiotemporal calcium activity patterns that regulate biophysical processes—such as convergent extension and apical constriction (i.e., the cellular morphogenesis process where neural plate cells contract their apical sides and bend the neural plate inward)—that are important for neural tube closure [75]. For example, in zebrafish and *Xenopus*, a slow rise in calcium in the dorsal ectoderm has been suggested to be critical for early neural induction by enabling crosstalk between several neuralizing signals (e.g., calcineurin, FGF/Erk and BMP) [20,40,70,72,76,77]. Furthermore, in *Xenopus,* this increased calcium has been demonstrated to upregulate arginine *N*-methyltransferase *xPRMT1b*, which induces expression of neural differentiation markers such as *zic3* and *N-tubulin* [78].

Batut et al., attempted to understand how *xPRMT1b*, a calcium-induced target gene, plays a role in neural induction [78]. To do so, they prepared open-face Keller explants in *Xenopus* embryos and imaged calcium activity using aequorin. Given that they did not have single-cell resolution, they measured the number of photons per second over time for the tissue visualized in each image. They found that *xPRMT1b* expression is correlated positively with calcium levels. They also found that overexpression of *xPRMT1b* induces the expression of neural genes like *Zic3.* Thus, they concluded that calcium levels could regulate neural fate through *xPRMT1b* expression regulation during gastrulation [78].

The frequency, duration, and regularity of different types of calcium activity have been suggested to regulate different biophysical and physiological processes during early neural development. For instance, in *Xenopus*, high frequency calcium activity in relatively isolated cells (i.e., spikes) is implicated in inhibitory fate specification of cells, and low frequency calcium activity has been implicated in excitatory fate acquisition [79]. Furthermore, wave-like multicellular activity propagating across several to hundreds of neighboring cells has been implicated in coordinating tissue movements during gastrulation and neural tube closure [39,70,80]. However, the calcium imaging and analysis techniques used to investigate the role of calcium in neural tube formation are inconsistently applied across different studies, which creates barriers for comparison of results between studies and may explain discrepancies in types and patterns of calcium activity implicated in neural tube formation across existing literature.

In a study attempting to characterize calcium transients during blastula formation and gastrulation, Akahoshi et al., imaged calcium activity in *Ciona* embryos during gastrulation using the genetically encoded calcium indicator (GECI) GCaMP6s; in particular, the study manually counted the number of calcium transients in calcium activity images for analysis [81]. In addition to imaging *Ciona* embryos during gastrulation, the study also imaged *Ciona* embryos at the neurula stage. At this stage, calcium transient images were also manually identified. The study results revealed that calcium transients were found in precursors of muscle cells in the late gastrula stage and were observed in neurogenic cells in the neurula stage. Akahoshi et al., also found that the average duration of calcium transients was 39 ± 4 s in neurogenic cells but 22 ± 4 s in the visceral ganglion. Because this was a characterization study, the study was unable to identify any causal effects of calcium on development [81].

In a separate study attempting to image calcium activity during convergent extension in gastrulation, Wallingford et al., prepared open-face DMZ explants of *Xenopus* embryos during the early gastrula stage [80]. In particular, the study used the calcium green dextran fluorescent reporter and a confocal imaging system to detect calcium activity. Because the study’s images were not able to capture calcium at a single cell resolution, it was unable to identify calcium spikes. However, they did observe calcium waves throughout convergent extension. In order to analyze its data, Wallingford et al., defined calcium activity as and analyzed ΔF/F0 values ranging from 1 to 3 and an F0 baseline value manually chosen from a region apparently not active with calcium. Importantly, the study did not apply a threshold cut off value. Wallingford et al., ultimately found that many calcium waves occurred in the dorsal region of explants during convergent extension and were often accompanied by a wave of contraction in the tissue. It also found that pharmacological inhibition of calcium signaling inhibited convergent extension without affecting cell fate. Thus, the study concluded that calcium waves must play an integral role during convergent extension [80].

Shindo et al., also attempted to image calcium activity during convergent extension [82]. Instead of Calcium Green Dextran, the researchers injected *Xenopus* embryos with the genetically encoded calcium indicator (GECI) GCaMP, along with membrane-RFP, a fluorescent protein marker for cell membranes. The study also involved creating open-face Keller explants and imaging calcium activity during convergent extension. Like Wallingford et al., Shindo et al., also manually quantified the number of calcium “flashes” (a term the study used to describe calcium transients in general). Despite having single cell resolution through membrane-RFP in the study’s images, this study did not differentiate between calcium spikes and waves. Overall, Shindo et al., found that inhibition of calcium flashes led to blocked cell polarization in the Keller explants and revealed that the purinergic receptor P2Y11 is required for calcium flashes and cell polarization [82].

In another study attempting to image calcium activity in mesodermal tissue during gastrulation, Hayashi et al., removed the ectoderm of *Xenopus laevis* embryos and conducted “cap-less” calcium imaging during gastrulation [83]. After injection of YC-Nano3GS mRNA at the four-cell stage, calcium activity was imaged using a spinning disk confocal microscope with 100–500 ms scanning every 15–120 s for 0.5–3 h. The study performed a background subtraction on its calcium time-series data by defining the background as the average intensity of an empty sample region. Additionally, calcium transients were defined as an increase in the ratiometric value of the YFP and CFP fluorescence of YC-nano greater than the moving median ratiometric value of the entire image. While this definition is both quantitative and objective, it requires the use of a specific type of calcium indicator, YC-Nano, which emits both YFP and CFP fluorescence. Thus, such an analysis would not work with single fluorescent value readout methods, such as the more common GECI, GCaMP. Nevertheless, Hayashi et al., found that calcium levels in the leading cells are markedly higher than those in the following and axial mesoderm cells during gastrulation. The study also found that inhibiting calcium activity decreased cell movement during gastrulation, leading to the conclusion that calcium signaling must play an important role during cell movement in gastrulation [83].

In contrast, Kreiling et al., used the calcium indicator Oregon green BAPTA-1 dextran and the calcium-insensitive Texas red dextran to conduct ratio-metric imaging during gastrulation [84]. In particular, the study used the MetaMorph 7.0 image analysis software to measure average green and red fluorescence intensities in the same regions of interest. It then calculated the ratio of fluorescence of Oregon green to Texas red, determining the calcium concentration using the formula [Ca^2+^] = K_d_(R − R_min_)/(R_max_ − R), where K_d_ = 620 nM, R_min_ = 0.7, and R_max_ = 4.1, ultimately presenting the calcium imaging data in terms of concentration [84]. However, Kreiling et al., did not explicitly define calcium pulses by describing baseline or signal threshold parameters used in image analysis. The study found that there is a distinct pattern of calcium with higher concentrations in the ventral margin and lower calcium concentrations in the dorsal margin. Additionally, it revealed that suppression of calcium signaling with thapsigargin created left-right asymmetries of the heart and brain organs. This supported the study’s conclusion that calcium signaling is important during gastrulation and regulates key processes for proper organogenesis. In particular, a limitation of the methodology used in the study is that ratio-metric imaging can make it difficult to image calcium activity at single cell resolution as at least three separate fluorescent proteins are needed to do so [84].

In addition, Papanayotou et al., attempted to determine the role of calfacilitin in neural induction by conducting calcium imaging in wild type or calfacilitin-MO chick embryos [85]. Specifically, the study used either Rhod-2 (imaged by conventional fluorescence with TRITC filters in rectangular or circular regions of interest), a mixture of Fura-Red and Fluo4 (acquiring the ratio between green and red fluorescence using conventional fluorescence microscopy), or Fura 2 (imaged using conventional fluorescence microscopy or confocal microscopy) to detect calcium activity. The study concluded that Rhod-2 was the most stable calcium indicator (despite its tendency to accumulate in the mitochondria) and was advantageous to use as it enabled combination with FITC-labeled morpholino or GFP-labelled constructs. One result of the study was its finding that loss of calcifacilitin led to a loss in calcium signaling, which can be rescued by ionomycin. The study also concluded that calcifacilitin is required for expression of *Geminin* and *Sox2*, implying its role in neural plate formation [85].

To study mechanisms that regulate apical constriction during neural tube closure, Suzuki et al., imaged calcium activity at stage 16 (neural fold stage) in *Xenopus* embryos using the calcium-sensitive R-GECO1.0 fluorescent reporter (at 20- or 40-s intervals for up to 6 h) and the calcium-insensitive EGFP fluorescent reporter (an injection control) [43]. To detrend the imaging data collected, Suzuki et al., performed a linear regression between R-GECO1.0 and EGFP intensities at each pixel, and then used residual values for subsequent analysis [43]. The study defined calcium transients as when the ratio between GECO1.0 and EGFP exceeded 1.25. Importantly, the study did not segment out individual cells; rather, objects were defined as 50 pixels large, with 1.33 microns per pixel. Of note, objects greater than 500 pixels were defined as multicellular calcium transients. The study found that calcium waves lasted up to 100 s and that waves spread at a speed of 3.3 ±0.94 µm/s on average. Additionally, Suzuki et al., found that multicellular transients happened one order of magnitude less frequently than single-cell transients. Furthermore, the study supports the hypothesis that calcium transients lead to F-actin remodeling and subsequent apical constriction and closing of the neural plate. Suzuki et al., also found that extracellular ATP and N-cadherin play a role in this apical constriction, supporting the conclusion that calcium transients are important modulators of the apical constriction process during neural tube closure [43].

In *Drosophila*, calcium has been implicated in dorsal closure (a similar process to vertebrate neural tube closure) (reviewed in [86]). To study the impact of calcium signaling in *Drosophila* dorsal closure, Hunter et al., used C2:GFP, a calcium-dependent fluorescent indicator, to image calcium activity during dorsal closure [87]. They compared embryos injected with GsMTx4, a specific modulator of mechanically gated ion channels which caused increases in cytosolic calcium, with control injected embryos. They found that embryos injected with GsMTx4 had increased actomyosin contractility. Interestingly, dorsal closure was blocked in a dose-dependent manner with injection of GsMTx4. This suggested to Hunter et al., that calcium plays an integral role in dorsal closure through the coordination of actomyosin contraction in a dose-dependent manner. Given the nature of their experiment, they did not have operational definition to identify calcium transients. They did, however, correct for photobleaching using ImageJ, subtracted the background from the image (defined as the mean pixel value in a non-C2:GFP-expressing cell) and applied a Gaussian blur. They then smoothed the image using a Savitzky–Golay filter [87].

In another study attempting to determine the role of calcium signaling during apical constriction in neurulation, Christodoulou and Skourides imaged stage 14 (late neural plate stage) *Xenopus* embryos through neural tube closure using the membrane-GFP cell membrane marker and the GECO-RED calcium indicator while imaging at a rate of 0.1 Hz [42]. Calcium transients were manually identified as either “calcium flashes” or calcium waves, and “calcium flashes” were further categorized as single cells, small group of cells (2–4 cells), or larger groups of cells aided by membrane-GFP cell membrane marker. However, the definition used to classify calcium transients as flashes or waves remains unclear [42].

Additionally, by using simultaneous tracking of the change in cell surface area, calcium activity time series, and expression levels of Lulu (an apical constriction inducing molecule), Christodoulou and Skourides showed that apical actin enrichment occurs within one minute after cell-autonomous and asynchronous calcium activity, but never before or during a calcium activity event [42]. Ultimately, Christodolou and Skourides concluded that apical constriction is largely driven by calcium transients followed by actin contractions during neural tube closure. Implications that cytosolic calcium activity levels influence cytoskeleton remodeling have also been offered by studies using other model systems, including neurulation in mice [88], vascular smooth muscle contraction in rabbits [89], wound closure in *Drosophila* pupal epithelium [90], and dorsal closure in *Drosophila* [87].

In a study attempting to determine the role of NMDA receptors in neural tube closure, Sequerra et al., imaged calcium activity in *Xenopus* embryos during neural tube closure beginning at stage 15 (late neural plate stage) [41]. GCaMP6s was used to visualize calcium activity, which was imaged at a rate of 0.1 Hz for 2 h. The study set a threshold for calcium transients at a fluorescent value equal to twice the noise. Additionally, Sequerra et al., cited a range of previous papers for further details on its calcium analysis methodology [91,92,93,94,95,96]. However, it was unclear what the study’s definition of noise (i.e., baseline) actually was following a review of these papers [79,92,93,95,96]. Nevertheless, Sequerra et al., found that neural plate cells exhibit calcium spikes that are in part regulated by glutamate signaling during neural tube formation. The study also found that NMDA receptors are important for neural tube formation and a loss of NMDA receptors results in neural tube defects [41].

Additionally, Abdul-Wajid et al., imaged *Xenopus* and *Ciona* embryos to study T-type calcium channels in neural tube closure [97]. In this study, *Xenopus* embryos were imaged at the neural plate stage at an imaging rate of 1 Hz and *Ciona* embryos were imaged at the neurula stage until the late tailbud stage at an imaging rate of 0.1 Hz. In particular, the study used the GCaMP3 GECI to image calcium activity in both organisms. Calcium transients were defined as an increase in fluorescence intensity greater than 30% of the baseline, which was defined as the mean fluorescence value of the region of interest over the entire time series. The study found that loss of T-type calcium channels upregulated *EphrinA-d* and *EPHA2* and that overexpression of *EphrinA-d* could lead to an open brain phenotype in *Ciona*. This led the researchers to conclude that T-type calcium channels and the *Ephrin* gene family play a role in neural tube closure. Because this study mainly focused on these specific genes and proteins, it did not have results or conclusions pertaining directly to calcium transients [97].

Spitzer et al., also imaged spinal neurons cultured from *Xenopus* embryos with cultures beginning at stage 15 (neural fold stage) and intact neural tube tissue using the Fura-2, Fluo-3, and Fluo-4 calcium indicators. Embryos were imaged at a rate of 0.1 Hz, and calcium transients were defined as fluorescent values greater than twice the standard deviation of the noise, which was not defined [98]. Spitzer et al., also defined filopodial transients as events exceeding 10% of baseline fluorescence in filopodia. As this was a methodological study, Spitzer et al., did not show results with these methods but instead recommended the methodology of imaging and analysis of calcium activity in neural tissue and cell culture described in the study [98].

Moreover, Zhao et al., revealed that hyperglycemia during embryonic development resulted in calcium signaling dysregulation and was correlated with neural tube defects. In this study calcium activity was imaged in C57BL/6J mice embryos using Fluo-4 and Indo-1 calcium indicators and treated embryos with mibefradil (a T-type calcium channel blocker) or verapamil (an L-type calcium channel blocker). It suggested that hyperglycemia-mediated calcium signaling primarily occurred through T-type calcium channels, as treatment of diabetic, pregnant mice during neural tube development with mibefradil (but not verapamil) resulted in significantly less neural tube defects. This study did not quantify calcium transients but rather simply determined the ratio between fluorescence under 405 nm and 485 nm wavelength light throughout the imaging period to measure the level of intracellular calcium levels, utilizing this ratiometric method as a measure of calcium signaling [99].

Finally, McMillen et al., developed a novel embryo imaging array to investigate the effects of bioelectric manipulation on image calcium signaling in multiple *Xenopus laevis* embryos during embryonic development [100]. To visualize calcium activity for imaging, *Xenopus laevis* embryos were microinjected with GCAMP6S mRNA at the four-cell stage and (if applicable) being microinjected with depolarizing ion channel *dnKir6.1* mRNA or hyperpolarizing ion channel *kv1.5* mRNA at the two-cell stage (for bioelectric manipulation, if applicable). This study found that between the neural tube closure and early tailbud stages of development, depolarization through *dnKir6.1* expression resulted in increased calcium signaling until later stages in development, where it began to result in calcium signal inhibition. Hyperpolarization through *kv1.5* expression similarly resulted in calcium pattern disruption albeit to a lesser degree than *dnKir6.1* expression. This study did not quantify calcium transients but instead manually scored for presence or absence of various calcium activity patterns (e.g., persistent high calcium patches, static calcium signals, and persistent high calcium spots) over time qualitatively during embryonic development [100].

The studies described have primarily investigated calcium signaling in gastrulation and neural tube closure. With gastrulation, many studies agree that there is increased calcium signaling during convergent extension and mesoderm invagination [80,82,83]. Inhibition of this calcium signaling can lead to aberrant tissue placement and development. During neural tube closure, many studies also agree that there is increased calcium signaling in the neural tissue, and inhibition of this calcium signaling leads to neural tube defects [41,42,43,97]. Thus, it appears that calcium signaling plays an integral role in tissue movement during development, particularly in gastrulation and neural tube closure, and also in apical constriction during neural tube closure. However, an important limitation of the studies investigating calcium’s role in neural induction is the lack of standardization of imaging and analysis techniques (e.g., data detrending and normalization methods, baseline definition, calcium transient threshold determination, spike and wave definition, and more) used to quantify calcium activity, making comparison of results between studies challenging.

## 5. Calcium Activity during Neuronal Subtype Formation and Neurotransmitter Phenotype Specification

As the embryo continues developing, the neural plate gives rise to distinctive anatomical regions. The neuroepithelial cells in the neural plate have the potential to undergo a terminal division to form differentiated cells or undergo a symmetric division to increase the size of the precursor cell pool first localized in the ventricular zone (VZ) in a single cell layer (known as the primary proliferative zone). Some cells in the precursor cell pool transform into radial glia (primary neural progenitor cells, NPCs) that ultimately give rise to a heterogeneous group of neurons that express different neurotransmitters and their receptors. The stable differentiated fate, that is the phenotype, of neurons is the identity of the neurotransmitters that they synthesize and release [101,102]. Various studies have been conducted to investigate the role of calcium activity in neural phenotype acquisition [103,104,105,106,107,108], but application of different analysis methods between studies (particularly when applying different baseline and threshold parameters) acts as a barrier to comparison of the results between studies (particularly for spike incidence, frequency, duration, and inter-event-interval), making it challenging to quantify the extent to which differing conclusions drawn between studies are the result of variations in image acquisition parameters and/or data analysis.

Several studies using various model systems have suggested that cells initially exhibit sporadic calcium activity but switch to their signature spontaneous activity upon differentiation. For example, using transgenic zebrafish embryos expressing pan-neuronal GCaMP6f and a microscopic framework monitoring long term calcium activity from neural plate to spinal circuit development stages, Wan et al., reported that motor neurons expressing cell-type specific marker mnx1 initially exhibit sporadic spontaneous activity, then exhibit periodic spontaneous activity eventually synchronizing globally by recruiting commissural interneurons as they continue to develop, then generate left-right ensembles required for early motor behavior in the developing spinal cord [109]. In contrast, other cell types (e.g., cerebrospinal fluid-contacting Kolmer–Agduhr neurons) in the same embryo continually exhibit sporadic spontaneous activity over time. To obtain these results, Wan et al., defined baseline activity as 20th percentile of a sliding time window for 61 time points and defined calcium activity as a ΔF/F trace that passed either of these two tests: (1) the trace contributed more than 0.3 to the factor loading in factor analysis or (2) the trace contained significant signal (*p* < 0.05) above the background noise as determined by a Kolmogorov–Smirnov test [109].

A separate cross-correlation analysis performed by Warp et al., between the sporadic activity traces that occur prior to ensemble formation (ipsilateral synchronization) in zebrafish embryos similarly shows that activity traces are rarely associated with any other cells [110]. Contrasting Wan et al., however, this study analyzed calcium activity by calculating the z-score of ∆F/F values, and defining activity as a rise in fluorescence (over a 1-s interval) that exceeds an empirically determined threshold subsequently refined using a nonlinear, least-squares fitting algorithm in MATLAB with a cubic spline, double exponential kernel, timing, and amplitude [81,110]. Akahoshi et al., similarly reported that visceral ganglion (VG) precursors show a rapid transition from slow sporadic activity to fast rhythmical Ca^2+^ activity with a duration of 22 ± 4 s and inter-activity time of 71 ± 6 s at the tailbud stage of development using intact Ciona embryos with GCaMP6s [81]. However, contrasting the previous two studies, Akahoshi et al., manually counted the number of Ca^2+^ transients while confirming observations with a ratiometric threshold method using FM4-64 plasma membrane marker intensity to determine a baseline [81].

In *Xenopus*, studies have shown that calcium activity patterns in developing spinal cord neurons are more variable. For example, by imaging the dorsal and ventral surfaces of the developing *Xenopus* spinal cord using Fluo-4 and using a paired *t*-test for comparison, Swapna and Borodonisky reported that the spiking incidence (percentage of cells exhibiting at least one spike in an imaging session) and frequency in the ventral versus dorsal neural tube differ significantly with ventral neurons exhibiting nearly two times greater numbers of spikes compared to the dorsal neurons and nearly two times greater numbers of spiking incidence. Furthermore, the spike shapes, durations, and inter-spike-intervals in activity traces were found to vary [95].

Similar results were reported previously by imaging dorsal sensory Rohon–Beard neurons (RB), dorsolateral interneurons (DLI), ventral motoneurons (MN), and ventral interneurons (VI) in an ex vivo preparation of developing spinal cord of *Xenopus laevsi* [79]. In this study by Borodinsky et al., by analyzing only the neurons that exhibit at least one spike over an hour (approximately 75 to 90% of total imaged cells do not exhibit spontaneous activity), the authors showed that these cell types exhibit their own signature activity patterns as they mature from early to late tail-bud stages (in this study, analysis was performed using three ranges of stages 20–22, 23–25, and 26–28). As the embryo continues to develop, RB neurons exhibit low and constant frequency spiking patterns (<2 spikes per hour), DLI exhibit monotonically increasing spiking frequency patterns (~2 to 6 spikes per hour), the frequency of spiking patterns of MN neurons step from low to high (~2 to 8 spikes per hour), and VI neurons exhibit high frequency activity patterns (~6 to 8 spikes per hour). While these neurons were not shown to exhibit activity traces as periodically as reported in zebrafish mnx1 positive neurons and VG cell in *Ciona* at tail bud stage, the cluster size of coactive neurons was reported to increase with maturity [79].

However, using a different species of Xenopus, *Xenopus tropicalis*, in similar in vitro and ex vivo preparation, Chang and Spitzer reported that spikes last for a shorter duration than in *Xenopus laevis*, and the trends of incidence and frequency across stages do not correlate in *Xenopus tropicalis* as they do in *Xenopus laevis* [111]. These observations indicate that while cells in the developing spinal cord exhibit sporadic activity patterns, some cells acquire rhythmicity and synchronize with other cells as they mature with timing of acquiring signature patterns of calcium activity differing between cell types. Using a similar calcium imaging technique as in Chang and Spitzer, Marek et al., found that calcium spike activity was positively correlated with cJun phosphorylation [112]. This led to increased *tlx3* expression and thus regulation of neurotransmitter specification in *Xenopus tropicalis* [112].

Because calcium activity during this phase of development is sporadic and complex (i.e., composed of a wide array of frequency ranges), different studies use different techniques (particularly with respect to applying baseline and threshold parameters) to analyze the calcium activity and draw conclusions about the role of calcium activity on phenotype acquisition. For example, in one study, Gomez and Spitzer imaged partially exposed *Xenopus* spinal cord labeled with fluo3 at the tailbud stage (stage-21–25/26), quantified calcium activity using measurements 15 min prior to release of case compound as a baseline, and defined spikes by applying a threshold of 150% of the baseline [113]. Furthermore, they considered the mean of fluorescent values less than 130% of the baseline as the noise. However, in a different study using similar spinal cord preparation and ratiometric imaging of filopodia, Gomez et al., defined a spike as a calcium activity event that exceeds 10% of baseline fluorescence, contrasting the definition of a spike used in the previously mentioned study. Furthermore, different from both aforementioned studies, to identify active cells in data obtained by imaging the ventral surface exposed neural tubes of *Xenopus* at stages 23–25 with Fluo4, Guemez-Gamboa et al., defined a spike as needing to satisfy these two conditions: (1) measured fluorescent value of calcium activity is 20% above twice the standard deviation of the baseline during the previous 10 min and (2) the rise time is complete within 5 s [114]. The use of different calcium analysis techniques (e.g., using different calcium spike definitions) across studies in existing literature creates challenges for comparing results between studies as it is unclear the extent to which using differential calcium analysis techniques impacts the conclusions drawn in the studies.

Furthermore, evidence for calcium activity-dependent neurotransmitter respecification (in contrast to neurotransmitter phenotype-dependent calcium activity) in developing nervous systems has also been accumulating steadily in literature. In other words, while initial expression of neurotransmitters is determined by genetic programs, calcium activity patterns have been implicated in this process [25,79]; reviewed in [102]. For example, Borodinsky et al., report that embryos exhibited higher numbers of glutamatergic (vGluT) and cholinergic (ChAT) neurons (excitatory neurons) when spiking frequencies in both the right and left sides of Xenopus embryos were suppressed by overexpressing human inward rectifier K^+^ channels (hKir2.1). Enhancing spiking frequency by overexpressing voltage-gated rat brain Na^+^ channels (rNav2aβ), the other hand, significantly reduced the number of neurons that expressed excitatory neurotransmitters (vGluT and ChAT) while increasing the total number of neurons that express inhibitory neurotransmitters (GABAergic and Glycinergic) [79]. Several subsequent studies further support how neurotransmitter switching is dependent on calcium activity and in particular underscore the importance of spike frequency on specifying neurotransmitter phenotype expression [94,95,114,115,116].

Bataille et al., reported that increased embryonic neural excitability proxied by calcium transients was correlated with the number of dopaminergic neurons in the subpallium but not in the olfactory bulb and that decreased embryonic neural excitability was correlated with the number of dopaminergic neurons in the subpallium and in the olfactory bulb [117]. This study did so by imaging for calcium activity visualized using the GCaMP6f indicator in zebrafish at 2–3 days post- fertilization for 30 min using the GCaMP6f indicator and treating the embryos with either veratridine (activation of a calcium channel, which increased embryonic neural), a cocktail of tetrodotoxin, ω-conotoxin, nifedipine, and flunarazine (calcium channel blockers, which decreased embryonic neural), or no treatment (control). This study defined calcium transients in two different ways: (1) calcium transients which were changes in fluorescence greater than two and a half standard deviations above the baseline and (2) changes in fluorescence greater than two standard deviations (2SD) above the baseline with a duration greater than three seconds. It was ultimately unclear which definition was used for their calcium activity analysis, and the method that they employed to determine their baseline for analysis was not explicitly stated [117].

Moreover, Munz et al., imaged calcium activity using two-photon microscopy in neurons of Rbp4-Cr KL100 mouse embryos [118]. This study used GCaMP6s as a calcium indicator to visualize calcium activity and quantified the total change of GCaMP6s fluorescence (%ΔF/F) (normalized per second) over time. In particular, a baseline for calcium activity analysis was determined by subtracting a rolling mean spanning 12.5 s (for E13.5) or 62.5 s (for all subsequent stages) from filtered calcium activity traces. Additionally, the study defined calcium events as at least 10%(ΔF/F)/second above baseline for more than 1.5 s. Calcium activity analysis revealed that in Rbp4-Cre somas and neurites, calcium events increased from E13.5 to E14.5, decreased from E14.5 to E15.5, remained low from E15.5 to E16.5, and increased from E16.5 to E17.5. Calcium activity further increased from E17.5 to E18.5 in somas but not in neurites, where activity reached a plateau. Munz et al., also suggested that voltage-gated sodium channels contribute to calcium activity increase in Rbp4-Cre neurons at E14.5 and E18.5 because calcium activity decreased upon treatment with tetrodotoxin (a voltage-gated sodium channel blocker) but not upon application of cortex buffer alone [118].

Overall, the studies investigating calcium’s role in neural phenotype acquisition are notably limited by the application of different analysis methods, which is a barrier to comparison of the results between these studies. This limitation creates challenges quantifying the extent to which differing conclusions drawn are the result of variations in calcium detection and analysis or true biological differences between studies.

## 6. Discussion

Compelling evidence from a variety of sources—including investigations focused on the expression of genes involved in calcium dynamics as well as observation of calcium fluxes in living embryos—has firmly established that calcium activity plays a critical role in mediating key processes throughout neural development in a wide range of organisms. While the experimental evidence emerging from gene expression studies is clearly important, in order to definitively establish the role of calcium activity during development, it is essential to demonstrate that functional calcium fluxes occur in vivo within the embryo. The goal of this review is to evaluate the body of literature that attempts to observe calcium activity in the developing nervous system in living embryos during embryonic stages. More specifically, we aim (1) to provide investigators with a range of alternatives for their own research; (2) to assess the current state of analysis techniques currently used in the field; and (3) most importantly, to provide suggestions for increasing the consistency, transparency, and reproducibility of calcium activity analysis techniques within the field.

Several conclusions emerge from the analysis provided in the previous sections. First, despite the diversity of analytical techniques, overall, an impressive array of studies using several different model systems (e.g., medaka fish, zebrafish, *Xenopus laevis*, and *Drosophila*) and calcium visualizers (e.g., aequorin, calcium-green dextran, fura-dextran, Oregon Green 488 BAPTA dextran, YC2.60 ratiometric calcium indicator, Texas red lineage marker, and GCaMP GECIs) have corroborated genetic evidence that calcium activity plays important roles in the early cleavage, blastula, and gastrula stages of development. All of the studies described in the review presented above document the presence of calcium activity in a key developmental process. Secondly, the studies employ a surprising wide diversity of analysis methods, making direct comparisons among the studies difficult to impossible. Furthermore, many studies provide insufficient detail for any type of comparison. Differing conclusions reached by the different studies (e.g., the role of calcium in furrow formation or the precise type of calcium of activity during neurulation) could be largely attributable to the significant variations in analysis techniques. Finally, despite the huge importance of calcium in so many critical developmental events, there are still relatively few studies that actually visualize calcium in living embryos; overall, compared to the hundreds of studies on expression of genes involved in calcium dynamics, in vivo whole embryo calcium imaging remains poorly studied.

Given the overall paucity of studies that image calcium activity during key developmental stages in vivo, the profound disparity in analysis methods, and the frequent lack of sufficient detail on the precise methods employed, a number of recommendations emerge from the analysis of the literature provided in this review. First, and perhaps most obviously, additional studies that employ in vivo calcium imaging at key points in neural development are needed in order to generate a comprehensive and reproducible picture of the role of calcium in neural development. This not only includes understudied stages of development (such as the stages in between gastrulation and neural tube closure), but the need for multiple groups to corroborate the observed calcium dynamic patterns. Studies that associated the expression of genes implicated in calcium dynamics with actual calcium activity imaging in whole, living embryos would be particularly valuable. Moreover, many of the studies are correlational rather than causal. While correlations between calcium activity and gene expression or a given phenotype represent an important first step, establishing causality by showing that changes in gene expression affect calcium activity and conversely how changes in calcium activity affect phenotype is critical to advance our understanding of the role of calcium activity in neural development. Future studies should aim to establish causation between calcium activity and developmental phenotypes by utilizing mechanical, optical, chemical, and biological calcium manipulation tools.

Second, specific methodologies for analyzing calcium activity, namely manual identification of calcium activity to analyze time-lapse images should be avoided. While it is possible to visually identify calcium transients using images acquired, this methodology is limited by its lack of quantitative and objective parameters for defining what a calcium transient is. Additionally, manual calcium transient detection methods are difficult to use for images that capture large areas of tissue (and in turn many cells). Thus, future efforts should be made to analyze calcium using quantitative ratio-metric methods rather than manually.

Third, studies that employ a calcium analysis method at single cell resolution are limited. While cells are large enough to easily be visualized during early cleavage stages, for late cleavage and blastula stages, without a membrane marker, the classification of calcium activity types into spikes or waves is greatly limited. It may be beneficial to re-conduct the studies lacking cellular resolution while adding cell membrane markers and more quantitative analysis methods to the experimental methodology. Additionally, future studies should aim to achieve cellular resolution of calcium activity so that more informative insights about calcium dynamics can be made. However, a consideration when analyzing calcium activity acquired at a cellular resolution is that grove cells appear smaller and more wedge-shaped compared to other cells in the confocal images as they undergo cell elongation along the apical-basal axis of the neural plate, which may result in discrepancies between activity counts analyzed using cellular boundaries compared to unit area boundaries [119]. Nevertheless, investigators should aim to identify calcium activity in specific cells that are accompanied with other genetic markers to establish specific cell identities.

Fourth, it is essential that there be more consistency and standardization across studies that analyze in vivo calcium activity in the developing nervous system. Other fields including microarray, qRT-PCR, and RNA-Seq, have made enormous strides by developing accepted standards for data acquisition and deposition. The field of calcium activity analysis could likewise benefit from implementing such approaches. This does not mean that every investigator should adopt the exact same analytical approach, but rather, at the minimum, all studies involving calcium activity analysis should be required to report detailed, explicit information on the imaging rates, background definition and subtraction, baseline definitions, normalization techniques, and calcium transient definitions (including thresholds) to analyze calcium signaling data. For example, application of different baseline and threshold parameters used to identify when a cell has calcium activity can impact spiking incidence, frequency, duration, and inter-event-interval of calcium activity measured. This strongly argues for investigators to employ multiple methods of analysis to their data to determine if they obtain similar conclusion. Does using a range of thresholds for background determination and subtraction or for defining a spike change the interpretation of the data? Additionally, having the raw calcium activity data freely and easily accessible to the community so that others can analyze the data with their approaches, or new emerging approaches would not only enhance reproducibility but also for a more rigorous comparison of validity and robustness of the various approaches and the degree to which the choice of analysis method dictates conclusions regarding calcium activity. Similar to the sequencing and gene expression fields, increased standardization and transparency will reveal novel insights into the key role that calcium activity plays in neural development.

## Supplementary Materials

The following supporting information can be downloaded at: https://www.mdpi.com/article/10.3390/biom14010138/s1, Supplementary Table S1: Table of experimental parameters used for analysis of in vivo calcium activity during embryogenesis [2,25,40,41,42,43,44,45,48,49,52,53,55,57,58,59,60,61,62,63,64,65,66,67,79,80,81,82,83,84,85,93,95,98,99,100,103,104,105,106,107,108,109,110,111,112,113,114,115,116,117,118,120,121,122].
